# Filling data analysis gaps in time-resolved crystallography by machine learning

**DOI:** 10.1063/4.0000280

**Published:** 2025-01-21

**Authors:** Justin Trujillo, Russell Fung, Madan Kumar Shankar, Peter Schwander, Ahmad Hosseinizadeh

**Affiliations:** 1 Department of Physics, University of Wisconsin-Milwaukee, 3135 N. Maryland Ave, Milwaukee, Wisconsin 53211, USA; 2 Department of Chemistry-BMC Biochemistry, Uppsala University, Husargatan 3, Uppsala 75237, Sweden

## Abstract

There is a growing understanding of the structural dynamics of biological molecules fueled by x-ray crystallography experiments. Time-resolved serial femtosecond crystallography (TR-SFX) with x-ray Free Electron Lasers allows the measurement of ultrafast structural changes in proteins. Nevertheless, this technique comes with some limitations. One major challenge is the quality of data from TR-SFX measurements, which often faces issues like data sparsity, partial recording of Bragg reflections, timing errors, and pixel noise. To overcome these difficulties, conventionally, large volumes of data are collected and grouped into a few temporal bins. The data in each bin are then averaged and paired with the mean of their corresponding jittered timestamps. This procedure provides one structure per bin, resulting in a limited number of averaged structures for the entire time interval spanned by the experiment. Therefore, the information on ultrafast structural dynamics at high temporal resolution is lost. This has initiated research for advanced methods of analyzing experimental TR-SFX data beyond the standard binning and averaging method. To address this problem, we use a machine learning algorithm called Nonlinear Laplacian Spectral Analysis (NLSA), which has emerged as a promising technique for studying the dynamics of complex systems. In this work, we demonstrate the power of this algorithm using synthetic x-ray diffraction snapshots from a protein with significant data incompleteness, timing uncertainties, and noise. Our study confirms that NLSA is a suitable approach that effectively mitigates the effects of these artifacts in TR-SFX data and recovers accurate structural dynamics information hidden in such data.

## I. INTRODUCTION

Determination of three-dimensional (3D) structures and dynamics of biological molecules at high spatial and temporal resolutions is one of the main goals of structural biology and biophysics. The pump–probe technique is one of the methods in time-resolved crystallography carried out using synchrotron radiation^1–3^ and XFELs.^4–7^ Despite the differences between these x-ray sources,^3,8^ both provide time series of x-ray diffraction patterns that need to be analyzed to extract the 3D structural dynamics of a given molecule. In this work, we will primarily focus on the analysis of data from TR-SFX experiments by XFELs.

**FIG. 1.**
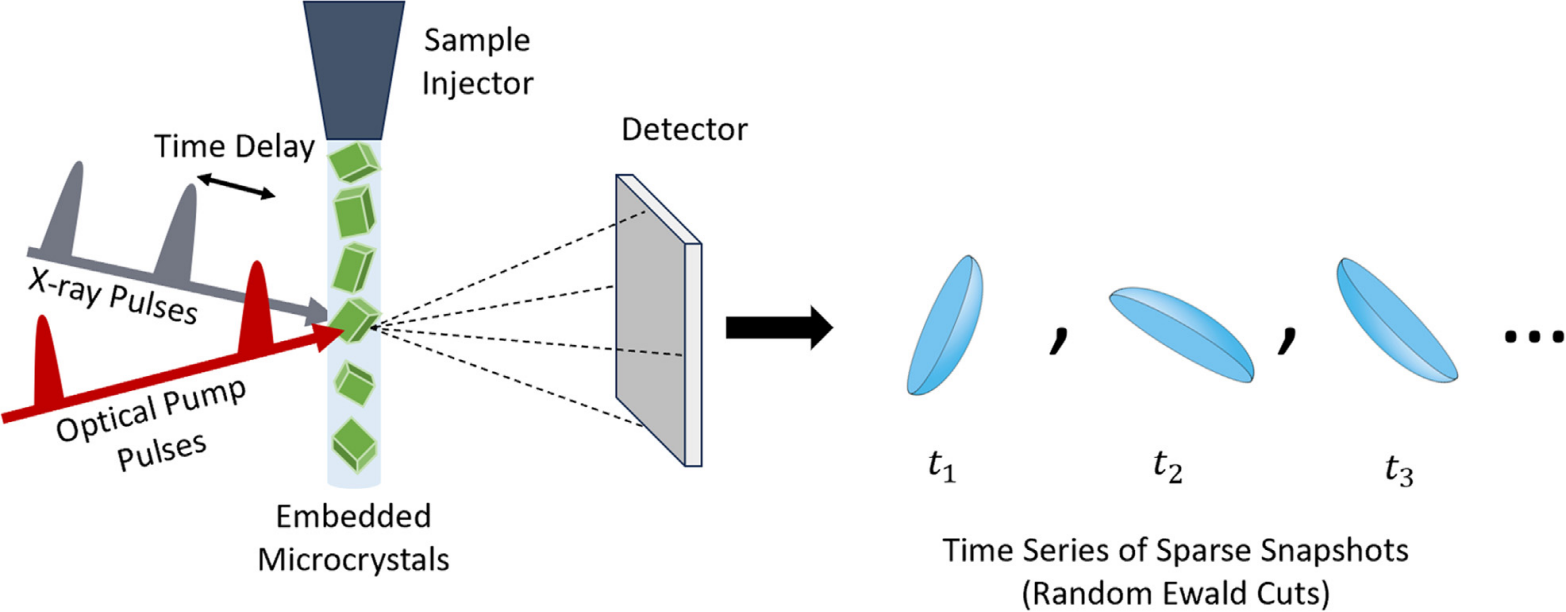
Schematic of conventional TR-SFX pump–probe experiments. Femtosecond x-ray pulses are delivered to a stream of microcrystals embedded in a liquid stream, which have been optically excited by a pump beam pulse. The resulting diffraction snapshots, which are essentially random cuts from the Ewald sphere, are recorded on a detector along with their corresponding delay times ( $t_{1},t_{2},\ldots$) and comprise a time series of diffraction data.

In conventional TR-SFX experiments, as illustrated in Fig. 1, microcrystals are embedded in a viscous liquid medium. This liquid containing the crystals is streamed through the x-ray exposure path. To study the dynamics of a protein in an optically excited state, pulses from an optical pump beam also periodically hit the stream of crystals, and the delay time between the optical excitation and the x-ray diffraction is recorded, while the x-ray diffraction patterns are collected on a detector.^9^ In a typical experiment, the collected diffraction patterns can easily number in the hundreds of thousands.^10^ This method of data collection differs from traditional x-ray crystallography experiments at synchrotron light sources, which are performed on larger crystals that are rotated through a wide range of angles to ensure that a complete diffraction pattern is collected.^5^ By contrast, the microcrystals in serial femtosecond crystallography (SFX and TR-SFX) experiments undergo negligible rotation since they are exposed to the x-ray beam for a very short amount of time.^11^ Moreover, the pulse duration of the beam is very short and has such high intensity that the sample is destroyed nearly immediately after diffraction has occurred.^9,10,12^ Due to the negligible rotation and short lifetime of each measured microcrystal, each of the collected diffraction “stills” or “snapshots” contains an incompletely recorded set of Bragg reflections for the crystal.^13,14^ Furthermore, there are effects from the energy bandwidth of the x-ray beam as well as minor structural irregularities of the microcrystals. This results in an effect known as partiality, which means that each Bragg spot in the snapshot was not fully measured.^10,11,15,16^ In addition to this data incompleteness factor, the experimental data also suffer from systematic and random noise,^12,13^ beam intensity variations,^10,15,17^ temporal instabilities (in the case of time-resolved experiments),^6,15,18^ and variability in other key parameters, such as size, orientation, and quality of crystals, detector response, sample injection, and so forth. In addition, the data are said to be very sparse, meaning the measured Bragg spots in a snapshot are too few to sufficiently describe the structure and dynamics of the system on their own. As a result, many data processing algorithms may break down.^19,20^ Therefore, to obtain more accurate structure and dynamics, it is crucial to identify and minimize these artifacts, which will lead to the efficient use of samples and decrease the experimental workload to obtain high-quality data.

Monte Carlo integration^13^ and data binning^4,6,21,22^ are established methods used by the crystallography community to mitigate data incompleteness. For a sufficiently high number of diffraction snapshots of randomly oriented microcrystals, Monte Carlo integration constructs a complete diffraction volume by averaging all snapshots together to a common intensity scale. In the absence of time-dependent structures, the spatial resolution improves as more snapshots are included in the analysis, and so a sufficiently large amount of data is needed to overcome data incompleteness.^13,16^ When searching for a time-resolved structure, as in TR-SFX, snapshots that fall within a set of time ranges are binned, and each bin is treated with the Monte Carlo averaging method individually. Consequently, a unique structure from each bin is produced, which allows for structural changes to be identified in time.^4,6,21,22^ However, this technique is crucially confined to determining, at best, a few discrete pictures of molecules far apart in time (on the order of 100 fs, to the authors' knowledge).^4,6,21–24^ Therefore, interesting and important dynamical phenomena happening in femtosecond timescales and below are not accessible via this approach.

The alternative way to deal with data incompleteness and timing errors is to use manifold-based methods.^18,25^ Interesting and important features of TR-SFX datasets can be uncovered by these machine learning approaches while simultaneously allowing for stochastic features to be filtered out.^25^ Commonly, methods such as singular value decomposition (SVD) and singular spectrum analysis (SSA)^26^ are deployed to analyze time series datasets. However, these linear methods are not relevant when dealing with nonlinear features in complex molecular systems. To address this problem, we use the Nonlinear Laplacian Spectral Analysis (NLSA)^27^ algorithm, which has successfully revealed the ultrafast structural dynamics of molecular systems that were previously challenging to quantify.^18,25^ As a complementary study to our previous work,^18^ here we further demonstrate and validate how NLSA handles the experimental challenges involved with serial femtosecond crystallography snapshots, such as data incompleteness (partiality and sparsity), as well as timing jitter in the case of time-resolved experiments. In the following sections, we first review the algorithm of NLSA. We then apply it to a set of synthetic x-ray crystallography diffraction patterns from a protein in its ground (dark) state, where the primary limitation is data incompleteness. We will compare the results with those obtained through Monte Carlo integration by CrystFEL,^28–30^ which is a conventional data analysis software in the field of x-ray crystallography. This is followed by the application of NLSA on time-resolved simulated data from the same protein, involving timing errors, data incompleteness, and noise. We then compare the reconstructed diffraction volumes at some selected time points with the original volumes. A similar comparison will be done by calculating difference electron density maps at selected time points.

## II. NLSA ALGORITHM

**FIG. 2.**
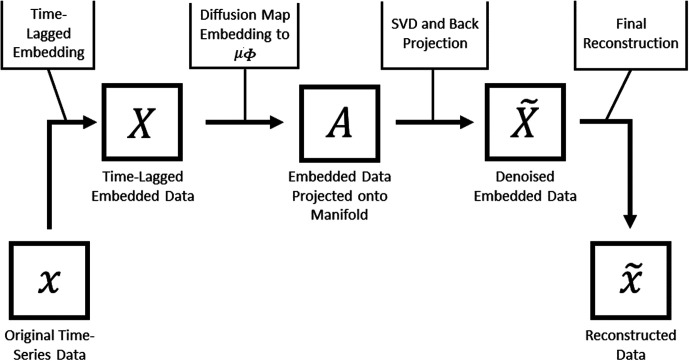
Schematic view of the NLSA algorithm. The original data x is time-lagged-embedded into a higher-dimensional space to give X. The curved manifold ( μΦ), which contains X is then found with the diffusion map algorithm. The data matrix X is projected onto the manifold to obtain A. Singular value decomposition of A and back projection of the leading modes to the embedded data space results in the matrix $\tilde{X}$. Diagonal averaging of $\tilde{X}$ gives the final reconstructed data $\tilde{x}$, which is a time series of diffraction volumes that are now full (i.e., no data incompleteness), as well as noise- and jitter-reduced.

The approach we employ is called Nonlinear Laplacian Spectral Analysis (NLSA). Here, we give a brief overview of this method. A schematic of the algorithm is shown in Fig. 2, and more details and applications can be found elsewhere.^18,25,27,31–34^ The NLSA process starts with a delay-coordinate embedding, which is a well-known method for the analysis of time-series data.^26,27,35,36^ The foundation of this technique is commonly known as Takens's theorem.^35^ This theorem states that a manifold containing the dynamical information of a system can be constructed from a series of time-lagged observations of the system. Consider a series of N measurements $\left\{x_{1},x_{2},\ldots ,x_{N}\right\}$ from a dynamical system, such as diffraction pattern snapshots with D pixels. A delay (time-lagged) embedded data matrix X is constructed such that each column (or supervector) corresponds to a concatenation of the original data vectors: $X_{t}={\left(x_{t},\;x_{t-\delta t},\ldots ,x_{t-(c-1)\delta t}\right)}^{T}$, where the embedding window (or concatenation order) *c* is the number of lagged copies of the original time series. Each $X_{t}$ is constructed by concatenating together original snapshot vectors $x_{t-i\delta t}$, where 0≤i≤c−1, in time sequential order so that each supervector contains *c* snapshots.

To extract the dynamical features of a system, one possible way to proceed is to apply singular value decomposition (SVD).^26^ However, in general, the output of standard SVD may not be relevant for systems with complicated nonlinear dynamics.^27^ To overcome this limitation, the nonlinear (“curved”) space manifold that the data matrix X occupies is identified through the nonlinear dimensionality reduction method known as diffusion map embedding.^19^ The X matrix is then projected onto this manifold and a matrix A is obtained as

A=XμΦ, (1)

where *Φ* and μ denote the leading dimensions and the Riemannian curvature of the curved space, respectively. Standard SVD is now applied to the matrix A so that

$$A=\sum_{m=1}^{K}U_{m}\;S_{m\;}\;V_{m}^{T},$$ (2)

where U and V are, respectively, the left and right singular vectors, and S are the singular values. Like the standard SVD, the first few modes with the largest singular values describe the most significant degrees of freedom in the data, and the remaining modes usually correspond to noise or other data artifacts. Therefore, a new matrix $\tilde{X}$ can be reconstructed by taking only the leading SVD modes and then projecting them back from the manifold onto the data space,

$$\tilde{X}=[\sum_{m=1}^{M<K}U_{m}\;S_{m\;}\;V_{m}^{T}]\Phi^{T},$$ (3)

where M<K is the number of selected modes. This is followed by averaging of the diagonal elements of $\tilde{X}$, which results in a reconstructed series $\left\{{\tilde{x}}_{i}\right\}$ of recovered information for each originally incomplete measurement. Note that in TR-SFX experiments, $x_{i}$ is a two-dimensional diffraction pattern with a pump–probe delay timestamp, and ${\tilde{x}}_{i}$ is a three-dimensional reconstructed diffraction volume at single time points. This means that the output of the NLSA approach can be a time series of 3D full diffraction volumes at high temporal resolutions.^18,25^

It is important to note that in standard data processing methods, the data complexities mentioned earlier in this paper are either ignored or handled through the binning and averaging approach. We make similar assumptions in the application of our NLSA algorithm. The main difference between NLSA and conventional analysis is that averaging is performed on a nonlinear manifold. NLSA does not require explicit binning and therefore does not suffer from loss of temporal resolution in time-resolved studies due to averaging binned data. This enables the algorithm to capture coherent structural changes that may occur in ultrafast timescales.^18^

## III. SIMULATING DIFFRACTION SNAPSHOTS

To compare NLSA to basic Monte Carlo averaging, we simulated dark state diffraction snapshots of photoactive yellow protein (PYP:PDB ID:5DH3).^4^ Here, a brief description of this procedure is given. More details of the simulation, as well as preprocessing and analysis of dark state data with NLSA are covered in the supplementary material.^48^ We used the *pattern_sim* function of the crystallographic data processing software CrystFEL^28,29^ (version 0.10.2). This program simulates diffraction snapshots when a user provides a PDB file, a full list of Bragg reflections, x-ray beam energy and size, microcrystal size, and desired noise levels. More information can be found in CrystFEL literature and the user manual for *pattern_sim*.^28–30^ For this work, the simulated x-ray beam energy was chosen to be 9.5 keV and have a radius of 1 μm. Each microcrystal was chosen to have dimensions in the range of 900–1100 nm along each side. The full reflection list for PYP^4^ used in the simulations was generated using CCP4^37–40^ (version 8.0). A total of 16,000 diffraction snapshots were simulated, and the noise was added to them as implemented by default in CrystFEL (version 0.11.0). This set of diffraction patterns was then indexed with the *indexamajig* function of CrystFEL to pair the simulated Bragg intensities with the appropriate Miller indices. To apply Monte Carlo merging to these data, the *partialator* function of CrystFEL (version 0.11.0) was used with the partiality model set to unity since we only want to perform basic Monte Carlo merging. The *partialator* function does compute an overall scaling factor and the Debye–Waller temperature factor for each crystal during the merging process. These scaling parameters were extracted from CrystFEL and applied to the unmerged data separately for use in NLSA. This way, the comparison between NLSA and Monte Carlo merging would be on data that have been suitably corrected with standard crystallographic considerations. A total of 15,865 diffraction snapshots were successfully indexed and scaled by CrystFEL. The resulting set of Monte Carlo merged reflections from CrystFEL's *partialator* function was used in the proceeding comparative analysis. The data input to the NLSA algorithm were the scaled but otherwise unmerged reflections.

**FIG. 3.**
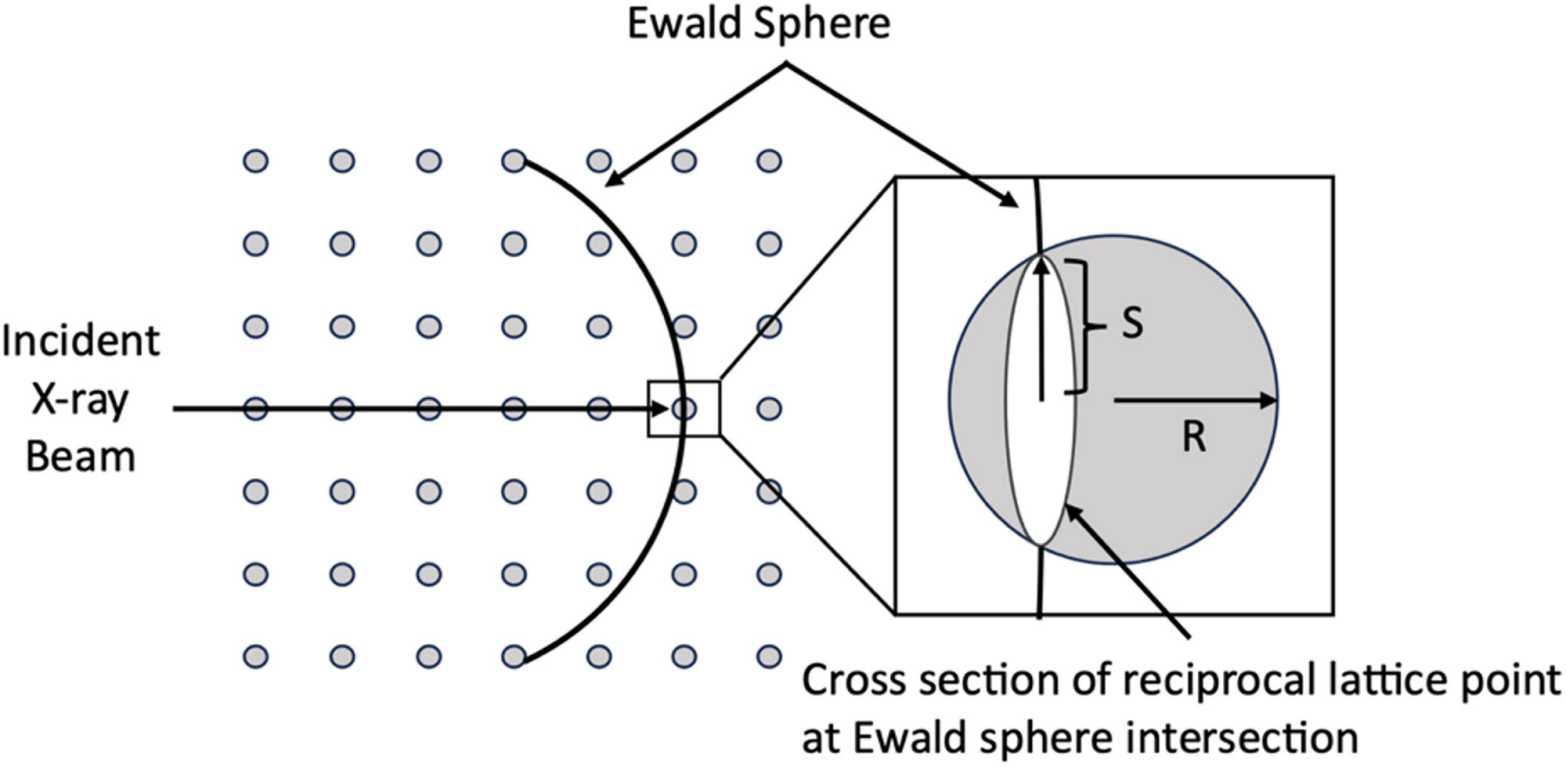
Schematic view of simulated data incompleteness and partiality. The enlarged area shows the intersection of the Ewald sphere in reciprocal space with the reciprocal lattice point. In this work, the reciprocal lattice point is approximated as a sphere of radius *R*, and the intersection of the Ewald sphere and the reciprocal lattice point is treated as a circle of radius *S.*

To evaluate the performance of NLSA on a time series of a biological molecule undergoing a dynamical process, we simulated a set of diffraction snapshots of optically excited PYP using the information from an experimental time series dataset obtained at the Linac Coherent Light Source (LCLS).^4,18^ In these simulations, randomly oriented Ewald cuts were taken from the full diffraction volumes (equally spaced about 70 attoseconds apart^18^) to incorporate the effect of the random orientation of the microcrystals as well as data incompleteness, as illustrated in Fig. 3. Partial reflections were calculated by modeling Bragg spots as spheres of radius R=1/(a/25) and using an x-ray beam energy of 9.5 keV. The factor of a/25 was chosen to result in the typical percentage of Bragg reflections observed in an actual experiment. The intersection of the Ewald sphere with each Bragg spot was approximated to be a circle of radius S≤R. This circular cross section was used to scale the Bragg spots down from their full intensities, thereby incorporating partiality. Finally, noise was added to each Bragg intensity according to a Gaussian distribution with a mean of zero and a standard deviation equal to the standard deviation of each set of Bragg intensities. This dataset, consisting of 87 141 snapshots, was then analyzed with NLSA as described in the Results section.

**FIG. 4.**
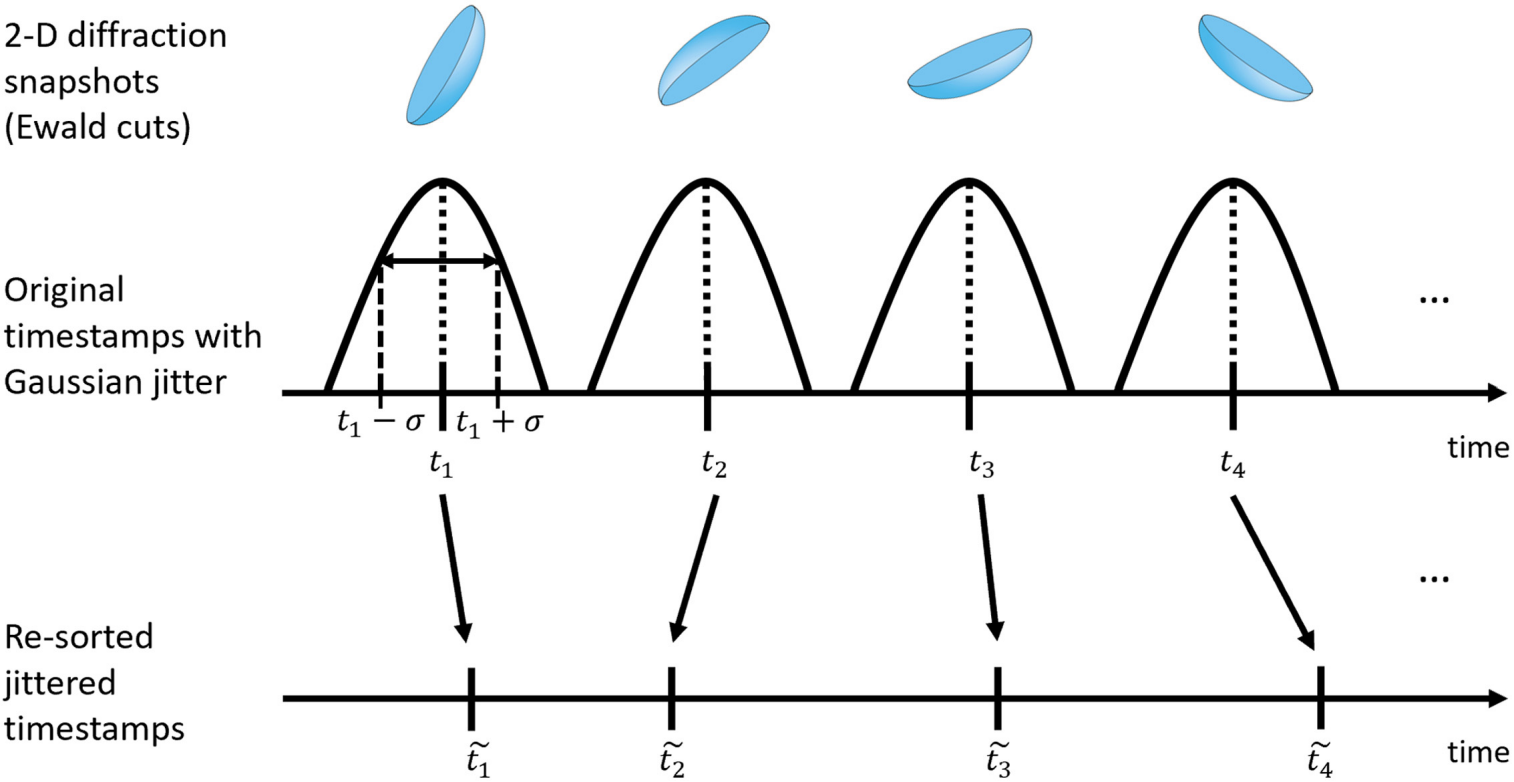
Schematic of simulating timing jitter. The simulated diffraction snapshots had an initial temporal ordering. The temporal ordering of the snapshots was then reshuffled to a degree based on the timing uncertainty observed under experimental conditions.^18,24^

To evaluate the performance of NLSA on a dataset affected by timing uncertainty, which is one of the main complications of data analysis in TR-SFX experiments, we introduced timing jitter into the simulated diffraction snapshots of excited-state PYP. This is illustrated in Fig. 4. We began with the synthetic dataset which did not include timing uncertainty. For each of these snapshots, a jitter offset time was randomly chosen from a Gaussian distribution with a standard deviation of 100 fs, which was chosen to be very similar to the reported uncertainty for the laser pulse timing apparatus.^18,24^ The original timestamps of the snapshots were then changed by the jitter offset amounts and the samples were re-sorted according to their new timestamps. The snapshots whose adjusted time stamps fell outside the original range of the un-jittered data were eliminated from consideration. The resulting dataset, consisting of 75 646 snapshots, was then analyzed with the NLSA algorithm.

## IV. RESULTS

**FIG. 5.**
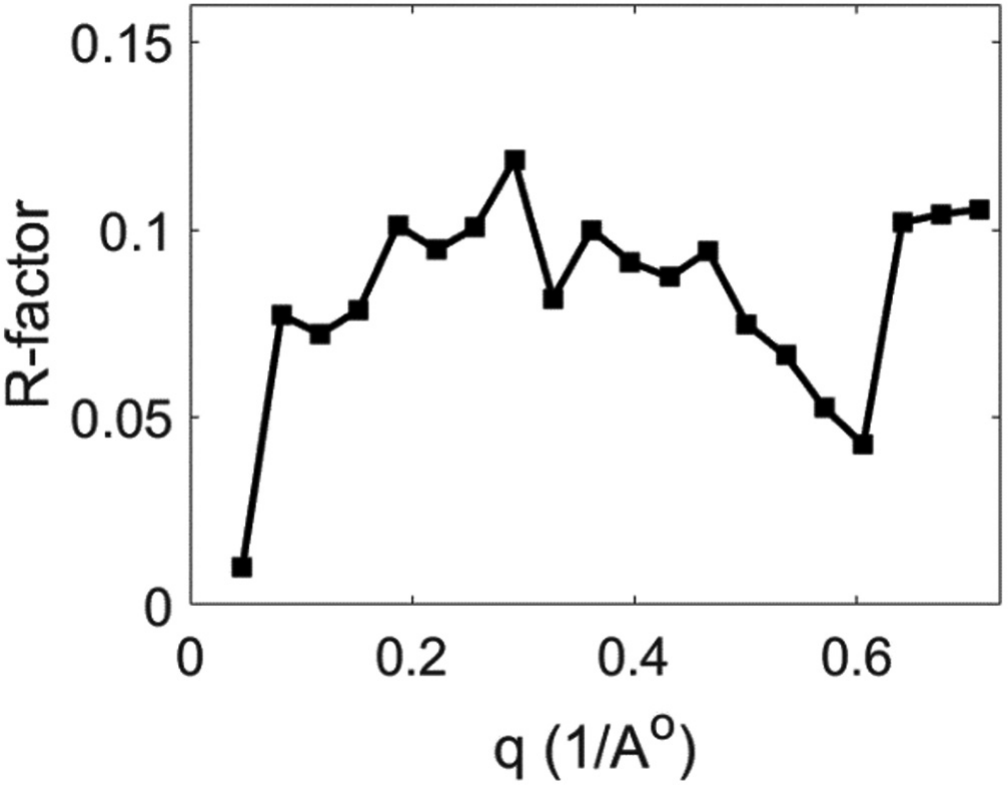
R-factor as a function of spatial resolution between NLSA-reconstructed data and Monte Carlo merged data for dark state PYP. The plot shows the R-factor remains well below 0.1 for most spatial resolutions. This confirms that NLSA performs the same as the well-established Monte Carlo merging method for resolving structural information from a system not undergoing dynamical processes.

The results of the NLSA algorithm on dark state data were compared to the results of Monte Carlo merging. In doing this, a baseline result about the capabilities of NLSA on SFX data compared to the well-established Monte Carlo method is obtained. To perform NLSA, a delay embedding parameter corresponding to the number of lagged copies of the original series, as well as the number of modes to use for final data reconstruction must be chosen, as introduced in the NLSA Algorithm section of this work. For the simulated dark state snapshots, an embedding parameter of c=2048 was chosen, and five modes were used in the reconstruction process. Based on our experience, this number of dimensions (modes) can sufficiently recover the diffraction volumes of dark data. Despite having only 15 865 snapshots in the dataset, this parameter combination successfully constructed a final series of full diffraction volumes. The series of reconstructed diffraction volumes output by NLSA was averaged together to produce a final diffraction volume to compare with the output of simple Monte Carlo merging. To quantify the comparison, both datasets were placed on the same numerical scale with zero mean and unit variance, and R-factors were calculated between the Monte Carlo merged results and the NLSA results. These R-factors were plotted for a range of spatial resolutions (q-shells). The results are shown in Fig. 5. The results of NLSA and Monte Carlo merging are very close, as shown by the fact that the R-factor is well below 0.1 for most spatial resolutions. This affirms that NLSA performs comparably to the Monte Carlo merging method when analyzing a system not undergoing complex dynamical processes. The success of this basic yet important comparison provides the clearance to proceed with testing NLSA on time-resolved data, which has the added difficulty of timing uncertainty.

To apply NLSA on the time series of 87 141 simulated diffraction snapshots of optically excited PYP, a delay embedding parameter of c=8192 was chosen, and the number of modes used in reconstruction was 10, which were the modes with the strongest singular values of NLSA. This combination was found to sufficiently handle the uncertainty in this time-resolved dataset. To quantify the results of the reconstruction, the Pearson correlation coefficient was calculated for each corresponding pair of reconstructed and original diffraction volumes. The Pearson correlation coefficient is a measure of linear correlation between the two datasets and is given by

$$r=\frac{\sum_{i=1}^{n}\left(I_{i}^{\text{orig}}-\bar{I^{\text{orig}}}\right)\left(I_{i}^{\text{NLSA}}-\bar{I^{\text{NLSA}}}\right)}{\sqrt{\sum_{i=1}^{n}{\left(I_{i}^{\text{orig}}-\bar{I^{\text{orig}}}\right)}^{2}}\sqrt{\sum_{i=1}^{n}{\left(I_{i}^{\text{NLSA}}-\bar{I^{\text{NLSA}}}\right)}^{2}}},$$ (4)

where $I_{i}^{\text{NLSA}}$ is the reconstructed intensity value from a certain pixel, and $\bar{I^{\text{orig}}}$ and $\bar{I^{\text{NLSA}}}$ are the average values of the original and NLSA pixel-level intensities, respectively.

**FIG. 6.**
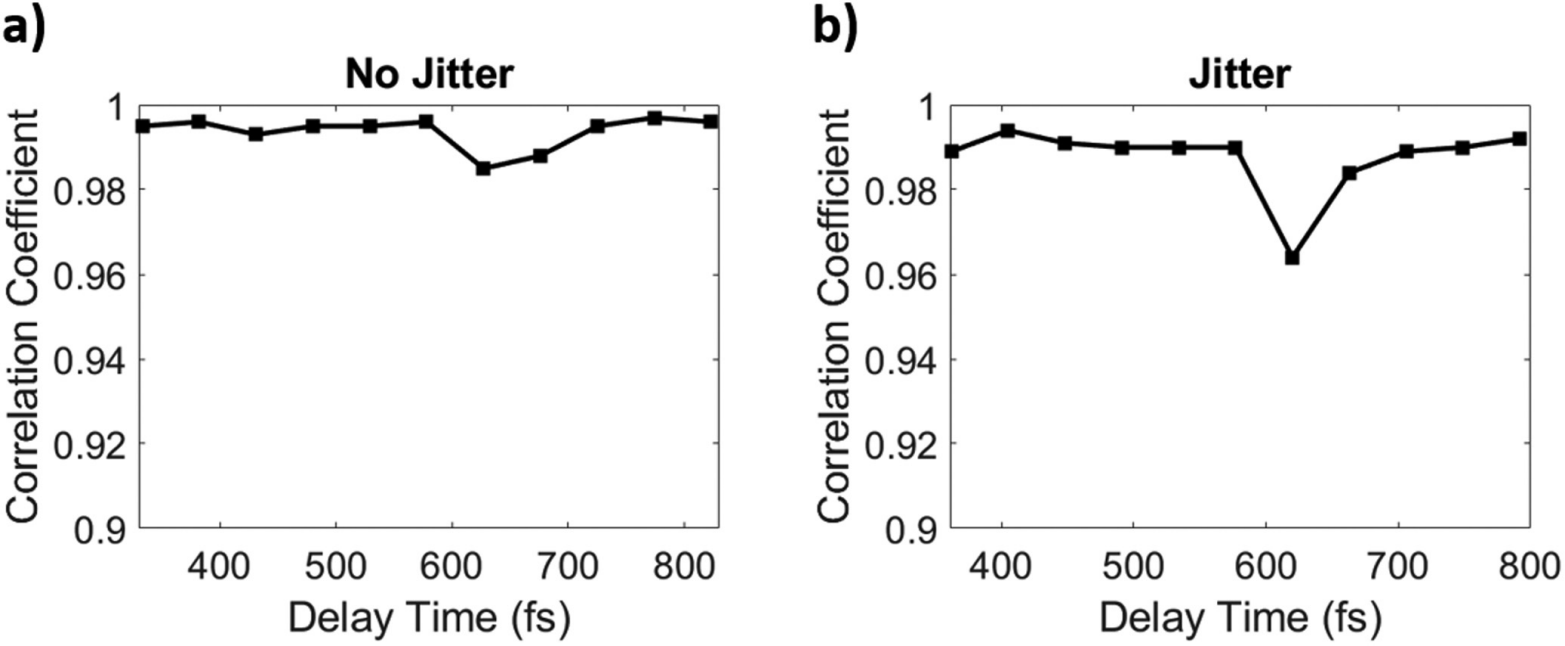
Plots of the Pearson correlation coefficients between each original and reconstructed diffraction volume. (a) Results for the case of no timing jitter. (b) Results for the case where timing jitter was included. In both cases, the correlation coefficient hovers just below 0.99, which indicates that NLSA is successful in structure determination even with significant data incompleteness and timing uncertainty.

For the simulated dataset which did not include timing jitter, the results of NLSA reconstruction were very successful. As shown in Fig. 6(a), the Pearson correlation coefficient for this case remains around a value of 0.995 for all volumes in the comparison and does not drop below 0.98. Therefore, despite the significant level of data incompleteness simulated in this dataset, the NLSA method produces a highly accurate reconstruction of the original data. This demonstrates the strength of NLSA when applied to data that are very sparse and imperfect, which was about 98 % incomplete compared to the full diffraction volumes that the snapshots were simulated from.

The same NLSA parameters and performance metric were used to evaluate the case of the 75 646 snapshots where timing jitter was applied. The Pearson correlation coefficients between the original and reconstructed intensities were calculated, and the results are shown in Fig. 6(b). As seen, the correlation coefficient is steady around 0.985 and does not drop below a value of 0.950. Despite the added timing uncertainty, NLSA was able to provide a faithful reconstruction of the original data.

**FIG. 7.**
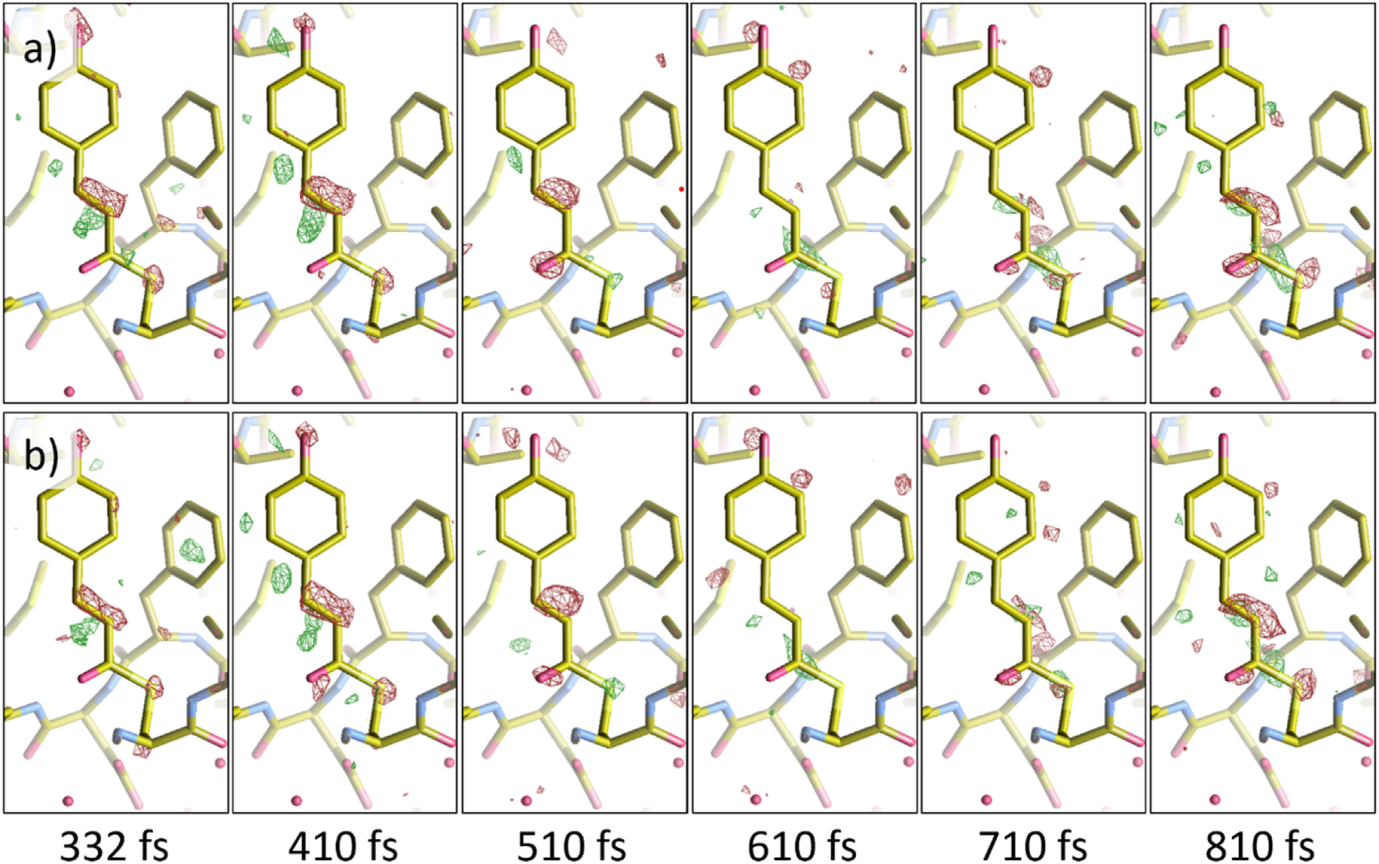
Comparing difference electron density (DED) maps calculated from (a) the original data (top row) to (b) those calculated from the NLSA-processed data without timing jitter (bottom row). The contour level of these maps is 3.0 RMSD. The average of the correlation coefficients between the DED maps for the delay times displayed here was 0.90.

**FIG. 8.**
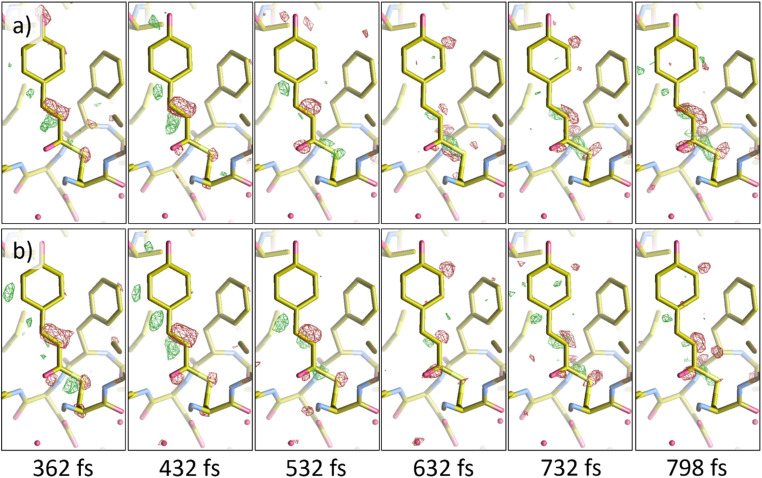
Comparing difference electron density (DED) maps calculated from the (a) original data (top row) to (b) those calculated from the NLSA-processed data with simulated timing jitter included (bottom row). The contour level of these maps is 3.0 RMSD. For these DED maps, the average of the correlation coefficients was 0.77, which is understandably lower than the case without significant timing uncertainty.

To further quantify the results of NLSA, difference electron density (DED) maps were calculated at several time points for both the non-jittered and jittered simulated datasets. These were compared with DED maps calculated from the original data at the same time points. The Pearson correlation coefficient between DED maps was used to quantify the results. To calculate a DED map, the structure factor amplitudes of a reference dark (i.e., not optically excited) dataset are subtracted from those of the excited dataset.^6^ The reference dark dataset used here was measured at the LCLS.^4^ The DED calculations were performed using various functions of CCP4,^37–46^ and the results were visualized using Coot^47^ (version 0.9.8.7). Figure 7 shows the results of the DED analysis for the case without timing jitter. As we are mainly interested in the chromophore of PYP, a mask with a 3 Å border around the chromophore region was applied to each DED map. For the delay times shown in Fig. 7, the average correlation coefficient was about 0.90. Figure 8 shows the results where timing jitter was introduced. The average correlation coefficient for the delay times shown in Fig. 8 was about 0.77. This drop in correlation coefficients is understandable given the introduction of significant timing uncertainty. Even so, NLSA reconstruction proved reliable in recovering useful structural information from a dataset with high levels of uncertainty.^48^

## V. CONCLUSIONS

In this work, we have demonstrated the application of a machine learning algorithm known as NLSA on simulated x-ray crystallography diffraction snapshots. NLSA was applied to dark state PYP snapshots simulated by CrystFEL, and to excited-state PYP snapshots simulated from experimental full diffraction volumes, with and without timing uncertainty. A baseline comparison with the long-standing Monte Carlo averaging method shows that NLSA successfully produces the same results for a non-excited system. Further, NLSA excels when analyzing time-resolved data. As the results show, NLSA is very effective at reconstructing time series of full diffraction volumes from simulated data, which suffers from incompleteness and timing uncertainty as well as noise. When no timing jitter was applied to the data, the Pearson correlation coefficients between the NLSA-reconstructed data and the original data remained above 0.98, confirming that NLSA is indeed a useful method for analyzing incomplete data with high sparsity. When timing jitter was applied to the simulated data, NLSA gave similarly satisfactory results, with the Pearson correlation coefficient remaining above 0.950 between the NLSA-reconstructed data and the original 3D volumes. The results in this paper have therefore demonstrated that NLSA can be confidently applied to TR-SFX experimental data, despite significant timing uncertainty and data sparsity, to determine crystal structures of proteins that are very close to the true structure. The DED map analysis clearly visualized and quantified the comparative features of original and simulated datasets. In the case without simulated timing jitter, the average correlation coefficient for the chromophore region of the DED maps between the original and NLSA-reconstructed volumes was 0.90. This average dipped to 0.77 when timing jitter was introduced but still shows that NLSA can recover meaningful information from a time series of data where the timing uncertainty is high. We conclude that this algorithm is a viable and valuable addition to the analysis routine of TR-SFX and other complex datasets. The results demonstrated in this paper support the more general idea that NLSA can be applied to other types of experiments where high-resolution dynamical information is obscured by high degrees of timing uncertainty and data incompleteness, as well as noise. Of course, more investigation is required to examine the impact of the other artifacts mentioned earlier on the functionality of NLSA, which we intend to pursue in the future using time-resolved crystallography data.

## Data Availability

The data that support the findings of this study are available from the corresponding author upon reasonable request.
